# Association between papillary thyroid cancer and *XRCC6* gene polymorphisms in the Turkish population

**DOI:** 10.55730/1300-0144.5902

**Published:** 2024-10-08

**Authors:** Egemen AKGÜN, Fadime MUTLU İÇDUYGU, Demet ŞENGÜL, Ebru ALP, Mehmet ALKANAT, Ayşegül ÇEBİ, Tuncer ÖZTÜRK

**Affiliations:** 1 Department of Medical Biology, Faculty of Medicine, Giresun University, Giresun, Turkiye; 2 Department of Medical Genetics, Faculty of Medicine, Giresun University, Giresun, Turkiye; 3 Department of Pathology, Faculty of Medicine, Giresun University, Giresun, Turkiye; 4 Department of Physiology, Faculty of Medicine, Giresun University, Giresun, Turkiye; 5 Giresun University Faculty of Health Sciences, Giresun, Turkiye; 6 Department of General Surgery, Faculty of Medicine, Giresun University, Giresun, Turkiye

**Keywords:** *XRCC6*, polymorphism, papillary thyroid cancer, PCR-RFLP, genotype-tissue expression

## Abstract

**Background/aim:**

To investigate the association between the rs2267437, rs5751129 and rs132770 polymorphisms of the *XRCC6* gene, which plays a role in repairing DNA double-strand breaks, and the risk of papillary thyroid carcinoma (PTC).

**Materials and methods:**

The study included 150 patients who had been diagnosed with PTC and 204 healthy controls. Genotyping of the SNPs was performed using polymerase chain reaction-restriction fragment length polymorphism (PCR-RFLP).

**Results:**

In the rs2267437 polymorphism, individuals with the GG genotype had lower risk of PTC than those with the wild-type CC genotype (p = 0.037, 95% CI: 0.19–0.96, OR: 0.67). The combined genotypes CG+GG were related to a reduced risk of PTC compared to the wild-type CC genotype (p = 0.023, 95% CI: 0.40–0.94, OR: 0.61) in the recessive model (GC+GG vs. CC). In addition, a query of the genotype-tissue expression (GTEx) database showed that the rs2267437 polymorphism may alter the expression level of *XRCC6* in whole blood (p = 0.0009) but not in thyroid tissue. There were no significant associations between the rs5751129 and rs132770 polymorphisms and PTC.

**Conclusion:**

This study demonstrated that rs2267437 polymorphism may have a protective effect against PTC in the Turkish population. However, the rs5751129 and rs132770 polymorphisms were not associated with the disease.

## 1. Introduction

Thyroid cancer is the most frequent endocrine cancer, with 586,202 new cases and 43,646 deaths in both sexes in 2020, according to GLOBOCAN 2020 (WHO global cancer statistics) data [1]. Furthermore, it is the second most frequently diagnosed cancer after breast cancer among women in Türkiye^1^ . There are four types of thyroid carcinoma according to their histopathological characteristics: papillary, follicular, medullary, and anaplastic. The most common of these is papillary thyroid carcinoma (PTC), which is responsible for 80% of all thyroid cancers. Although the causes of thyroid carcinogenesis are not fully understood, prominent risk factors include exposure to ionizing radiation and iodine deficiency/excess. [2,3]. However, differences in cancer development between individuals exposed to the same environmental factors suggest a genetic predisposition [4].

Some mutagens cause DNA damage, such as double-strand breaks (DSBs), because of their ability to break chemical bonds. Two main mechanisms, known as homologous recombination and nonhomologous end joining (NHEJ), are used to repair DNA DSBs [5,6]. Defects in these DNA repair mechanisms can result in genomic instability and may ultimately contribute to the development of cancer. Ku70 (*XRCC6*), one of the genes involved in the NHEJ mechanism, encodes a 70 kD protein. X-ray cross complementing group 6 (XRCC6), a component of DNA-dependent protein kinase (DNA-Pk), is required to recognize and bind to DNA DSBs [7]. Alterations in the expression of this protein can lead to carcinogenesis by reducing the repair capacity of the NHEJ repair mechanism. Several studies have indicated that variations in genes, which are related to the NHEJ mechanism, may cause a predisposition to many types of cancer [8–12]. The promoter region is a critical site for regulating transcription and maintaining mRNA stability. Many single nucleotide polymorphisms (SNPs) have been identified within the *XRCC6* promoter sequence. Among these polymorphisms, rs2267437, rs5751129, and rs132770 are found in the *XRCC6* gene promoter region and are very close to the binding sites of the transcriptional regulators responsible for proper transcription. Although the association of the *XRCC6* gene with cancer has been studied in some cancers, such as hepatocellular carcinoma [11], breast [13], oral [14], and colorectal [15] cancers, no research has been conducted to assess the relationship between thyroid cancer and *XRCC6* gene polymorphisms.

Hence, the aim of this study was to investigate the association between thyroid cancer and three selected polymorphisms (rs2267437, rs5751129, rs132770) in the *XRCC6* gene.

## 2. Materials and methods

### 2.1. Study population

This study was approved by the Ordu University Clinical Research Ethics Committee with the number 2/2016/8. The study protocol was conducted in accordance with the Declaration of Helsinki. The participation of all individuals in the study was voluntary and they were asked to sign informed consent forms. The study included 150 individuals with thyroid cancer as the patient group and 204 healthy individuals with no history of cancer as the control group. The diagnosis of thyroid cancer was made histopathologically by the Pathology Laboratory of Giresun University, Giresun Training and Research Hospital. It was ensured that none of the cancer patients were related to each other. In addition, individuals with a history of other cancers and those who had received previous treatment were not included in the study. The control group was chosen from patients attending the hospital for regular health assessments. Those with a thyroid-related disorder and those diagnosed with cancer were not included in the study.

### 2.2. Genotyping

Three milliliters of venous blood was taken from the study participants and stored at −20 °C until DNA extraction. DNA extraction was carried out using a high-purity PCR template preparation kit, which was commercially available, and in line with the manufacturer’s instructions (Roche Diagnostics, Mannheim, United Kingdom). The genotyping of *XCRR6* gene polymorphisms was performed using the PCR-RFLP method. A Bio-Rad T-100 thermal cycler (Bio-Rad Laboratories, Inc., Hercules, CA, USA) was used to perform the PCR reaction in a total volume of 50 mL. Primer sets were designed using Primer3 online software (National Center for Biotechnology Information (NCBI), Bethesda, MD, USA). The primer sequences used in the PCR reactions in the 5’-3’ direction were as follows: forward GGACCCACGGTTGTGTGAGATT, reverse CTGTGGAGACACTGGCGAAT for rs5751129 (T-991C); for rs2267437 (G-57C) forward CCTCCATCCCTTCCCCTTCCCCTTGAA, reverse AGCTCTACGTGTACGACCTG; for rs132770 (G-31A) forward AGAAGCTGGTTGGGGGGAGTGT, reverse TAACGGCCCGCTTACCTTTG. The PCR reaction mixture was prepared as 0.2 mM of each dNTP, 2.5 mM of MgCl_2_, 1U of Taq polymerase, and 0.5 mM of each primer. For the rs5751129 and rs132770 primers, a 35-cycle PCR was performed consisting of 5 min of initial denaturation at 95 °C, followed by 30 s of denaturation at 94 °C, 30 s of primer annealing at 55 °C, 45 s of chain extension at 72 °C, and 5 min of final chain extension at 72 °C. For the rs2267437 primer, a 35-cycle PCR was performed consisting of 5 min of initial denaturation at 95 °C, followed by 30 s of denaturation at 94 °C, 30 s of primer annealing at 58 °C, 45 s of chain extension at 72 °C, and 5 min of final chain extension at 72 °C.

The *Dpn*II restriction endonuclease was used to genotype the T-991C polymorphism. The presence of a C nucleotide in the region of the SNP provided a recognition site for the *Dpn*II enzyme. Thus, the PCR product with a total length of 152 bp was separated in the presence of the C-form into two fragments of 94 and 58 bp. To genotype the G-57C polymorphism, a total PCR product of 288 bp was treated with *Hae*II restriction endonuclease enzyme. As a result of the enzyme digestion, two fragments of 175 and 113 bp were obtained from the PCR product due to the presence of the G form in the SNP region. To obtain the genotypes of the A-31G polymorphism, a 240 bp PCR product was mixed with *Mnl*I enzyme. The restriction enzyme cleaved the PCR sequence into two fragments of 180 and 60 bp in the presence of the A-form. One unit of restriction endonuclease enzyme was used for all the enzyme cuts with a reaction time of 2 h at 37 °C. For visualization of the PCR and enzyme cut products, 3% agarose gel with ethidium bromide was used for the gel electrophoresis. Evaluation of the loaded gels was performed using the Gel Doc XR image system (Bio-Rad Laboratories, Inc.).

### 2.3. Statistical analyses

Differences in the clinical characteristics (age, sex) between the PTC and control groups were determined using the chi-squared test. The chi-squared test was used to assess the goodness of fit of the Hardy-Weinberg equilibrium (HWE) of the SNPs. Comparisons of the genotypic distributions between the patients and controls were performed using either the Pearson’s chi-squared or Fisher’s exact tests (when the expected value was less than five). The risk of PTC conferred by *XRCC6* polymorphisms was assessed using the odds ratio (OR) and 95% confidence interval (95% CI). To analyze the relationship between the genotypes and clinical features, χ2 tests were used for the categorical variables and one-way analysis of variance was used for the continuous variables. p < 0.05 was considered statistically significant. All the statistical analyses were performed using IBM SPSS Statistics for Windows 20.0 (IBM Corp., Armonk, NY, USA). The genotype-tissue expression (GTEx) database was used to evaluate the relationship between the rs5751129 polymorphism and *XRCC6* expression in different tissues [16].

## 3. Results

The study population comprised 354 individuals, of whom 150 had PTC and 204 were healthy. The clinicopathological parameters of the study are summarized in Table 1. No statistically significant differences in age or sex were found between the PTC and control groups (p > 0.05). The mean age of the individuals in the PTC and control groups was 52.04 ± 11.57 and 52.39 ± 10.03 years, respectively (p > 0.05). Most of the patients in the PTC group had been diagnosed with stage T1 cancer (79.3%). None of these patients had been diagnosed with stage T4. When the histological subtypes were examined, follicular PTC was found in 59.3% of the PTC patients. The frequency of extrathyroidal spread in the PTC group was 9.3%.

Genotype and allele frequencies of the rs2267437, rs132770, and rs5751129 polymorphisms in the PTC and control groups are shown in Table 2. There was no significant association between the rs5751129 polymorphism and PTC when comparing the PTC and control groups (p = 0.32). Similarly, no significant association was observed between the rs132770 polymorphism and PTC (p = 0.409). On the other hand, a significant association between the rs2267437 polymorphism and PTC was observed (p = 0.047). Those with the homozygous GG genotype had a reduced risk of developing PTC compared to those with the wild-type CC genotype (p = 0.037, 95% CI: 0.19–0.96, OR: 0.43). In the recessive model (GG+CG vs. CC), the combined genotypes GG+CG were associated with a decreased risk of PTC compared to the wild-type CC (p = 0.023, 95% CI: 0.40–0.94, OR: 0.61). When the allelic frequencies of the rs2267437 polymorphism were evaluated, the G allele may have had a significant protective effect against PTC in the study groups (p = 0.016, 95% CI: 0.49–0.93, OR: 0.67). The expression quantitative trait loci (eQTL) data from the GTEx database showed no association between the rs2267437 genotypes and *XRCC6* expression levels in thyroid tissue (Figure A), whereas the *XRCC6* expression increased in the presence of the wild-type C allele in whole blood (p = 0.0009) (Figure B).

When the genotype distribution in the patient group was compared with the clinical characteristics (multifocality, extrathyroidal extension, tumor diameter, histological type, and primary tumor), no statistically significant association was observed between the three polymorphisms and these characteristics (p > 0.05). However, although not statistically significant, multifocality was observed more frequently in those with the GG (9% for multifocality negative, 3.3% for multifocality positive) and CG+GG (51.7% for multifocality negative, 44.3% for multifocality positive) genotypes compared to the CC genotype (48.3% for multifocality negative, 55.7% for multifocality positive) of the rs2267437 polymorphism.

The distribution of all SNP frequencies obtained in the control group was compatible with HWE. However, in the PTC group, the frequency of rs2267437 was compatible with HWE, while the frequencies of rs5751129 and rs132770 were not. This deviation was unlikely to be the result of an error in genotyping, as the reliability of the results was increased by genotyping a randomly selected sample a second time. Deviation from the HWE is not always caused by genotyping errors. Other possible causes include genetic factors, such as chance or genetics (e.g., heterozygote advantage, population admixture/substructure, inbreeding, or copy number variations) [17–19].

## 4. Discussion

The aim of this study was to investigate whether three different SNPs in the *XRCC6* gene promoter region have any contribution to the development of PTC. The results showed that the G-57C (rs2267437) SNP may have a protective role against the risk of developing PTC in the Turkish population, whereas the T-991C (rs5751129) and A-31G (rs132770) polymorphisms were not associated with the disease.

The association between the *XRCC6* SNPs and different types of cancer has been investigated in the literature. However, the results seem to be contradictory (Table 3) [11,14,20–28]. For example, some studies performed in Taiwanese populations with different types of cancer (lung, oral, nasopharyngeal, renal cell, hepatocellular, and gastric cancers) declared no significant association between the rs2267437 polymorphism and these cancers [11,14,20,21,27,29]. Similarly, a multicenter study conducted in the Chinese population found that the association between breast cancer and the rs2267437 polymorphism was not statistically significant, but it was reported that this polymorphism increased the risk of breast cancer in estrogen receptor-negative/progesterone receptor-negative individuals [30]. Conversely, two different studies in the Chinese population showed that this polymorphism increased the risk of renal cell and esophageal squamous cell carcinomas [22,23]. A study by Willems et al. [28] in a European population likewise suggested that rs2267437 is a risk allele for breast cancer and that long-term estrogen exposure in individuals further increases the risk of cancer. In contrast to these studies, Fu et al. [13] associated the homozygous wild genotype of the rs2267437 polymorphism with an increased risk of breast cancer. Their data were similar to those obtained in the current study. There is insufficient information about the effect of the rs2267437 polymorphism on gene expression. The eQTL analysis in the GTEx database revealed that the rs2267437 polymorphism may alter the expression level of *XRCC6* in whole blood. The data in the present study suggest that this polymorphism may change the expression pattern of the *XRCC6* gene in PTC and therefore, carriers of the G allele are associated with a reduced risk of developing the disease. The results herein were also compared with data obtained from the UK Biobank (UKB) and FinnGen databases. Contrary to the current study, the UKB and FinnGen databases showed no significant relationship between the rs2267437 polymorphism and PTC (p > 5 × 10^−8^). The participant recruitment strategies of the studies may have had an effect on these different results. For example, the number of individuals with thyroid cancer included in the study in the UKB was relatively low and did not only consist of PTC patients. Additionally, there was no information in the UKB database indicating that individuals with different types of cancer were not included in the control group. In FinnGen, on the other hand, the fact that the individuals included in the study were from the Finnish population, which is a relatively closed society, may have been a factor in the different results.

In the present study, when the clinical features and genotypes were compared, no statistically significant difference was observed between the groups, but multifocality was found more frequently in patients with the rs2267437 polymorphism and GG and CG+GG genotypes. This result supports the finding that the rs2267437 polymorphism may be protective in thyroid cancer. In a study with a larger sample size, the association between multifocality and the rs2267437 polymorphism may demonstrate statistical significance.

In studies conducted in Chinese and Taiwanese populations, a significant association was found between the rs5751129 polymorphism and gastric, nasopharyngeal, hepatocellular, lung, oral, and renal cell cancers, whereas no association was found between the rs132770 polymorphism and these cancers in the same studies [11,14,20,21,25,27,30]. However, in a study conducted in the Chinese population, the minor allele of the rs132770 polymorphism was reported to be protective against renal cell carcinoma [31]. Different than these studies, the results of the current study for the rs5751129 polymorphism are consistent with a study conducted in the Iranian population reporting that no statistically significant association was found between breast cancer and the rs5751129 polymorphism [26].

The conflicting results of studies that have evaluated the relationship between the aforementioned *XRCC6* polymorphisms and cancer may be due to the fact that different mechanisms are involved in the development of different types of tumors and the Ku70 protein plays various roles in different processes of carcinogenesis. Additionally, the incompatible results may have been caused by differences in the sample sizes and the patients not having similar risk factors and ethnicity.

This study had some limitations, such as the moderate sample size and lack of an expression study. However, the variant allele frequencies of all the polymorphisms in the control group were compatible with the frequencies in the European population registered in the NCBI. This shows that there was no selection bias in the selection of individuals constituting the study group in terms of the genotype distributions.

## 5. Conclusion

This is the first study to investigate the relationship between PTC and these three polymorphisms. The results indicated that the rs2267437 polymorphism may have a protective effect against the risk of developing PTC. However, no significant association was found between the rs5751129 and rs132770 polymorphisms and PTC. Studies investigating the relationship between PTC and *XRCC6* polymorphisms in different populations, especially the Turkish population (in order to compare the results herein, regardless of ethnicity), will allow a more accurate comparison of the current results and the relationship between these polymorphisms and the development and progression of PTC will be more clearly elucidated. In light of future information on gene–environment interactions, data from such studies may contribute to our understanding of thyroid cancer.

## Figures and Tables

**Figure:**
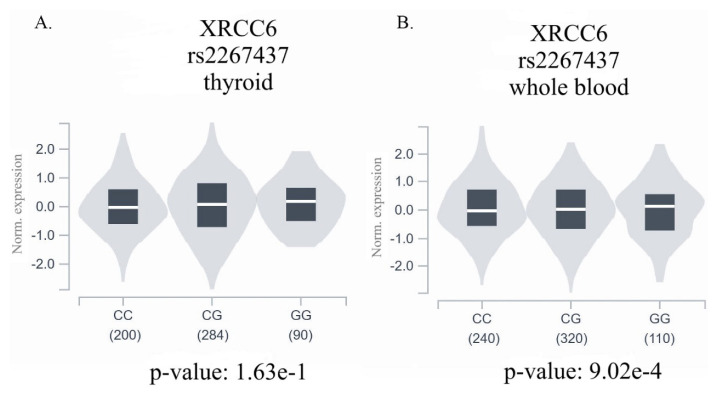
Effects of the rs2267437 genotype on the thyroid and whole blood *XRCC6* expression level. **A**. The presence of the SNP had no effect on the expression of *XRCC6* in the thyroid tissue (p = 1.63 × 10^−1^). **B**. The *XRCC6* expression increased in the presence of the wild-type C allele in whole blood (p = 9.02 × 10^−4^).

**Table 1:** Comparison of the PTC and control groups based on the selective characteristics.

Parameters		Patients n = 150	Controls n = 204	p-value
Age, years				
	≥45	111 (26%)	142 (74%)	0.37
	<45	39 (74%)	62 (28.9%)	
Sex				
	Female	120 (80%)	145 (71.1%)	0.56
	Male	30 (20%)	59 (28.9%)	
Histological type				
	Classical PTC	27 (18%)		
	Follicular PTC	89 (59.3%)		
	Classical-follicular PTC	9 (6%)		
	Unknown	25 (16.7%)		
Primary tumor				
	T1	119 (79.3%)		
	T2	25 (16.7%)		
	T3	6 (4%)		
Tumor size, cm				
	≥1	52 (34.7%)		
	1–2	53 (35.3%)		
	<2	45 (30%)		
Tumor location				
	Right lobe	51 (34%)		
	Left lobe	40 (26.7%)		
	Both lobes	47 (31.3%)		
	Isthmus	12 (8%)		
Multifocality				
	No	91 (60.3%)		
	Yes	59 (39.3%)		
Extrathyroidal extension				
	No	136 (90.7)		
	Yes	14 (9.3%)		

**Table 2:** Genotype and allele distribution of the SNPs in the PTC and control groups.

Genotypes	Patients (%) n = 150	Controls (%) n = 204	χ2 p-value	OR (95% CI)	p-value
T-991C (rs5751129)			0.320		
TT	80 (53.3%)	119 (58.3%)		1^a^	
TC	67 (44.7%)	77 (37.7%)		1.29 (0.84–2.00)	0.243
CC	3 (2%)	8 (3.9%)		0.56 (0.14–2.17)	0.533
TC+CC	70 (46.7%)	85 (41.6%)		1.22 (0.80–1.87)	0.349
Alleles			0.633		
C	227 (75.7%)	315 (77.2%)		1^a^	
T	73 (4.3%)	93 (22.8%)		1.09 (0.77–1.55)	0.633
G-57C (rs2267437)			0.047*		
CC	77 (51.3%)	80 (39.2%)		1^a^	
CG	63 (42%)	100 (49%)		0.65 (0.42–1.02)	0.061
GG	10 (6.7%)	24 (11.8%)		0.43 (0.19–0.96)	0.037*
CG+GG	73 (48.7%)	124 (60.8%)		0.61 (0.40–0.94)	0.023*
Alleles			0.016*		
C	217 (72.3%)	260 (63.7%)		1^a^	
G	83 (27.7%)	148 (36.3%)		0.67 (0.49–0.93)	0.016*
G-31A (rs132770)			0.409		
GG	85 (56.7%)	123 (60.3%)		1^a^	
GA	62 (41.3%)	73 (35.8%)		1.23 (0.79–1.90)	0.355
AA	3 (2%)	8 (3.9%)		0.54 (0.14–2.10)	0.532
GA+AA	65 (43.3%)	81 (39.7%)		1,16 (0.76–1.78)	0.493
Alleles			0.751		
C	230 (77.2%)	319 (78.3%)		1^a^	
A	68 (22.8%)	89 (21.7%)		1.06 (0.74–1.52)	0.751

* p < 0.05.

OR, odds ratio; CI, confidence interval; 1^a^ reference genotype/allele.

**Table 3:** Characteristics of the literature related to the *XRCC6* rs2267437, rs5751129, and rs132770 polymorphisms.

	Ethnicity	Cancer type	Sample size (patients/controls)	rs2267437 p < 0.05	rs5751129 p < 0.05	rs132770 p < 0.05
Chang [11]	Asian	Renal cell carcinoma	92/580	No	Yes	No
Wang [23]	Asian	Renal cell carcinoma	620/623	Yes	-	-
Wang [31]	Asian	Renal cell carcinoma	620/623	-	-	Yes
Fu [13]	Asian	Breast cancer	254/379	Yes	-	-
Yu [30]	Asian	Breast cancer	1039/1040	No	-	-
Rajaei [26]	Caucasian	Breast cancer	407/395	-	No	-
Willems [28]	Caucasian	Breast cancer	169/119	Yes	-	-
Hsu [21]	Asian	Hepatocellular carcinoma	298/298	No	Yes	No
Li [25]	Asian	Hepatocellular carcinoma	675/667	No	-	No
Tsai [20]	Asian	Nasopharyngeal carcinoma	208/416	No	Yes	No
Huang [24]	Asian	Nasopharyngeal carcinoma	176/352	No	Yes	No
Bau [14]	Asian	Oral cancer	318/318	No	Yes	No
Li [22]	Asian	Esophageal cancer	117/132	Yes	-	-
Yang [27]	Asian	Gastric cancer	136/560	No	Yes	No
Hsia [29]	Asian	Lung cancer	358/716	No	Yes	No

## Footnotes

1 International Agency for Research on Cancer (2021). Population fact sheets: Türkiye [online]. Website https://gco.iarc.fr/today/data/factsheets/populations/792-turkey-fact-sheets.pdf [accessed 04 January 2023].

## References

[1] Sung H Ferlay J Siegel RL Laversanne M Soerjomataram I Global cancer statistics 2020: GLOBOCAN estimates of incidence and mortality worldwide for 36 cancers in 185 countries CA: A Cancer Journal for Clinicians 2021 71 3 209 249 10.3322/caac.21660 33538338

[2] Feldt-Rasmussen U Iodine and cancer Thyroid 2001 11 5 483 486 10.1089/105072501300176435 11396706

[3] Schlumberger M Cailleux A-F Suarez HG de Vathaire F Irradiation and second cancers. The thyroid as a case in point Comptes Rendus de l’Académie des Sciences-Series III-Sciences de la Vie 1999 322 2–3 205 213 10.1016/s0764-4469(99)80045-6 10196674

[4] Czene K Lichtenstein P Hemminki K Environmental and heritable causes of cancer among 9.6 million individuals in the Swedish family-cancer database International Journal of Cancer 2002 99 2 260 266 10.1002/ijc.10332 11979442

[5] Featherstone C Jackson SP DNA double-strand break repair Current Biology 1999 9 20 R759 R761 10.1016/s0960-9822(00)80005-6 10531043

[6] Kanaar R Hoeijmakers JH van Gent DC Molecular mechanisms of DNA double-strand break repair Trends in Cell Biology 1998 8 12 483 489 10.1016/s0962-8924(98)01383-x 9861670

[7] Chiruvella KK Liang Z Wilson TE Repair of double-strand breaks by end joining Cold Spring Harbor perspectives in Biology 2013 5 5 a012757 10.1101/cshperspect.a012757 23637284 PMC3632057

[8] Bau D-T Fu Y-P Chen S-T Cheng T-C Yu J-C Breast cancer risk and the DNA double-strand break end-joining capacity of nonhomologous end-joining genes are affected by BRCA1 Cancer Research 2004 64 14 5013 5019 10.1158/0008-5472.CAN-04-0403 15256476

[9] Chiu C-F Wang H-C Wang C-H Wang C-L Lin C-C A new single nucleotide polymorphism in XRCC4 gene is associated with breast cancer susceptibility in Taiwanese patients Anticancer Research 2008 28 1A 267 270 18383855

[10] Han J Colditz GA Samson LD Hunter DJ Polymorphisms in DNA double-strand break repair genes and skin cancer risk Cancer Research 2004 64 9 3009 3013 10.1158/0008-5472.can-04-0246 15126335

[11] Chang W-S Ke H-L Tsai C-W Lien C-S Liao W-L The role of XRCC6 T-991C functional polymorphism in renal cell carcinoma Anticancer Research 2012 32 9 3855 3860 22993329

[12] Özgöz A Hekimler Öztürk K Yükseltürk A Şamlı H Başkan Z Genetic variations of DNA repair genes in breast cancer Pathology and Oncology Research 2019 25 107 114 10.1007/s12253-017-0322-3 28983784

[13] Fu Y-P Yu J-C Cheng T-C Lou MA Hsu G-C Breast cancer risk associated with genotypic polymorphism of the nonhomologous end-joining genes: a multigenic study on cancer susceptibility Cancer Research 2003 63 10 2440 2446 12750264

[14] Bau D-T Tseng H-C Wang C-H Chiu C-F Hua C-H Oral cancer and genetic polymorphism of DNA double strand break gene Ku70 in Taiwan Oral Oncology 2008 44 11 1047 1051 10.1016/j.oraloncology.2008.02.008 18487076

[15] Balinska K Wilk D Filipek B Mik M Zelga P Association of XRCC6 C1310G and LIG4 T9I polymorphisms of NHEJ DNA repair pathway with risk of colorectal cancer in the Polish population Polish Journal of Surgery 2019 91 3 15 20 10.5604/01.3001.0013.1030 31243170

[16] Lonsdale J Thomas J Salvatore M Phillips R Lo E The genotype-tissue expression (GTEx) project Nature Genetics 2013 45 6 580 585 10.1038/ng.2653 23715323 PMC4010069

[17] Li B Leal SM Deviations from Hardy-Weinberg equilibrium in parental and unaffected sibling genotype data Human Heredity 2009 67 2 104 115 10.1159/000179558 19077427 PMC2798818

[18] Cockerham CC Group inbreeding and coancestry Genetics 1967 56 1 89 10.1093/genetics/56.1.89 6035597 PMC1211496

[19] Weir B Hill W Cardon L Allelic association patterns for a dense SNP map Genetic Epidemiology: The Official Publication of the International Genetic Epidemiology Society 2004 27 4 442 450 10.1002/gepi.20038 15543640

[20] Tsai C-W Shih L-C Chang W-S Hsu C-L He J-L Non-Homologous End-Joining Pathway Genotypes Significantly Associated with Nasopharyngeal Carcinoma Susceptibility Biomedicines 2023 11 6 1648 10.3390/biomedicines11061648 37371742 PMC10296066

[21] Hsu C-M Yang M-D Chang W-S Jeng L-B Lee M-H The contribution of XRCC6/Ku70 to hepatocellular carcinoma in Taiwan Anticancer Research 2013 33 2 529 535 23393345

[22] Li K Yin X Yang H Yang J Zhao J Association of the genetic polymorphisms in XRCC6 and XRCC5 with the risk of ESCC in a high-incidence region of North China Tumori Journal 2015 101 1 24 29 10.5301/tj.5000206 25702660

[23] Wang W Pan X Huo X Yan F Wang M A functional polymorphism C-1310G in the promoter region of Ku70/XRCC6 is associated with risk of renal cell carcinoma Molecular Carcinogenesis 2012 51 S1 E183 E190 10.1002/mc.21914 22593040

[24] Huang C-Y Tsai C-W Hsu C-M Shih L-C Chang W-S The role of XRCC6/Ku70 in nasopharyngeal carcinoma International Journal of Oral and Maxillofacial Surgery 2015 44 12 1480 1485 10.1016/j.ijom.2015.06.008 26149939

[25] Li R Yang Y An Y Zhou Y Liu Y Genetic polymorphisms in DNA double-strand break repair genes XRCC5, XRCC6 and susceptibility to hepatocellular carcinoma Carcinogenesis 2011 32 4 530 536 10.1093/carcin/bgr018 21304054

[26] Rajaei M Saadat I Omidvari S Saadat M Association between polymorphisms at promoters of XRCC5 and XRCC6 genes and risk of breast cancer Medical Oncology 2014 31 4 1 4 10.1007/s12032-014-0885-8 24615008

[27] Yang M-D Wang H-C Chang W-S Tsai C-W Bau D-T Genetic polymorphisms of DNA double strand break gene Ku70 and gastric cancer in Taiwan BMC Cancer 2011 11 1 1 5 10.1186/1471-2407-11-174 21575261 PMC3111404

[28] Willems P De Ruyck K Van den Broecke R Makar A Perletti G A polymorphism in the promoter region of Ku70/XRCC6, associated with breast cancer risk and oestrogen exposure Journal of Cancer Research and Clinical Oncology 2009 135 1159 1168 10.1007/s00432-009-0556-x 19219618 PMC12160212

[29] Hsia T-C Liu C-J Chu C-C Hang L-W Chang W-S Association of DNA double–strand break gene XRCC6 genotypes and lung cancer in Taiwan Anticancer Research 2012 32 3 1015 1020 22399625

[30] Yu LX Liu LY Xiang YJ Wang F Zhou F XRCC5/6 polymorphisms and their interactions with smoking, alcohol consumption, and sleep satisfaction in breast cancer risk: A Chinese multi-center study Cancer Medicine 2021 10 8 2752 2762 10.1002/cam4.3847 33734613 PMC8026916

[31] Wang W Gao Y Yan F Wang M Hu F Association of Ku70 A-31G polymorphism and risk of renal cell carcinoma in a Chinese population DNA and Cell Biology 2012 31 7 1314 1320 10.1089/dna.2011.1540 22455395

